# Two decades of research on the role of diet in Alzheimer’s disease (2003–2023): a bibliometric and visual analysis based on CiteSpace

**DOI:** 10.1186/s41043-024-00503-9

**Published:** 2024-01-17

**Authors:** Wanyin Xu, Zhengyanran Xu, Yi Guo, Jing Wu

**Affiliations:** 1 Department of Nutrition, The First Affiliated Hospital of Zhejiang Chinese Medical University (Zhejiang Provincial Hospital of Chinese Medicine), Hangzhou, Zhejiang Province People’s Republic of China; 2 Department of Neurology, Epilepsy Center, The Second Affiliated Hospital of Zhejiang University School of Medicine, Hangzhou, People’s Republic of China

**Keywords:** Alzheimer’s disease, Diet, Nutrient, Bibliometric, CiteSpace, Visual analysis

## Abstract

**Background:**

In recent years, the impact of diet on Alzheimer's disease (AD) as a modifiable lifestyle has attracted widespread attention. We aimed to elucidate the current research status, frontiers, and research trends regarding the role of diet in AD over the past two decades through CiteSpace.

**Methods:**

Studies related to AD and diet that were published from January 1, 2003, to June 30, 2023, were retrieved via the Web of Science Core Collection. We imported the study data into CiteSpace for visual analysis of countries, institutions, co-authors, and co-occurring keywords.

**Results:**

A total of 922 relevant studies were included in our analysis, which found Nikolaos Scarmeas was the most prolific author (13 studies, 1.41%). The results also indicated that USA and Columbia University were the country and institution with the highest number of publications, with 209 (22.67%) and 23 (2.49%), respectively. The keywords that had a burst in the past four years were neuroinflammation, AD, tau, association, and beta.

**Conclusion:**

Talent exchange and regional cooperation are recommended in this study field. The results indicate that the effectiveness of various dietary patterns and mechanisms of dietary interventions using biomarkers and supplementation with refined nutrients will be the main research trends in the future.

## Background

Alzheimer's disease (AD) is the most common cause of dementia, characterized by deteriorating cognitive and social function as well as increasing dependency, which seriously affects quality of life and even shortens life span [1]. With the increase in the global aging population, the home-care pressure and economic burden due to dementia (represented by AD) will continue to escalate. Although its pathological changes cannot be reversed, available interventions and care can improve the symptoms of AD [2] and patients’ quality of life. A reasonable diet is one of the key factors in the prevention of age-related cognitive impairment [3]. Existing evidence has revealed that some nutrients (e.g., folate, flavonoids, vitamin D, and certain lipids) and food types (e.g., seafood, vegetables, and fruits, and possibly moderate alcohol and caffeine use) are associated with the protection of cognitive function in the elderly [4]. A review of Japanese longitudinal studies [3] showed that a nutritionally balanced diet containing a range of nutrients and foods was more effective than a diet with a single nutrient or food in preventing dementia. Compared with drug therapy, specific dietary patterns not only have potential value in promoting brain health, but also save medical costs [5]. Therefore, it is imperative to focus on diet in AD.

The relationship of AD with nutrients, foods, and dietary patterns has been studied for decades, with respect to genes, pathology, symptoms, treatment, and prevention [5–8]. Most of the scientific publications are observational studies and reviews, and there are few clinical trials. Moreover, current research has only explored certain aspects of the role of one or more foods and dietary patterns in AD [4, 9, 10], and there is no comprehensive overview of this research field over time that has identified hotspots and trends.

The purpose of the present study was to identify countries and institutions that have made significant contributions to the field and to identify and discuss hotspots and trends in research on dietary intervention and AD through the analysis of co-authors, co-cited references, keywords, and keywords with citation bursts, using CiteSpace [11]. CiteSpace is a Java-based application for bibliometric analysis that has the advantages of timely discovery and visualization of cutting-edge content in a research area, which can help one point to the direction of future research [12]. Therefore, we used this visual analysis tool to clarify the current status of studies on diet and AD and to predict the direction of future research.

## Methods

### Web of science core collection (WoSCC) search and data collection

**Fig. 1:**
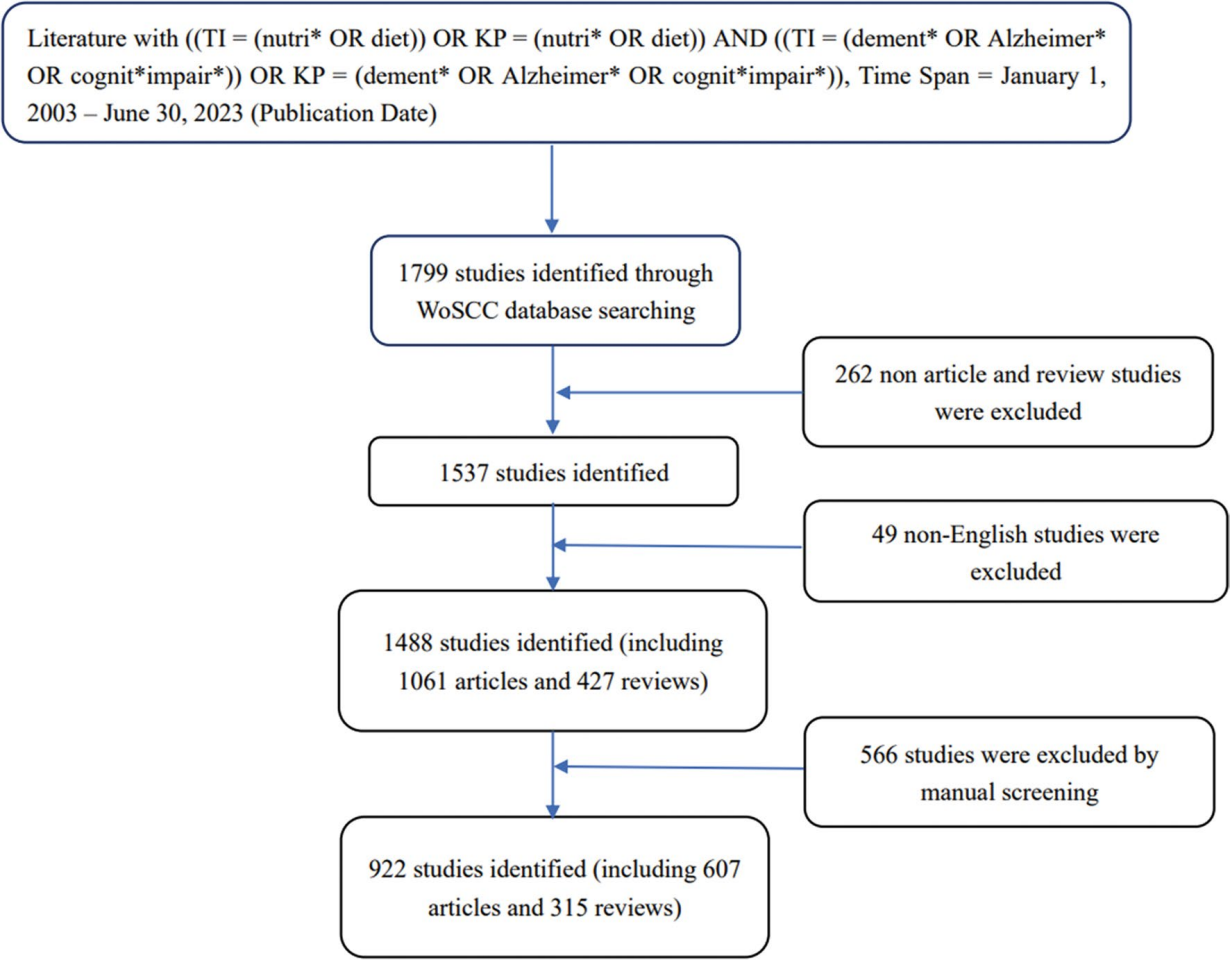
Flowchart of literature screening process

We chose to use bibliometric methods to analyze the research status in the field of diet and AD, in order to avoid distortion and bias due to subjective information filtering as much as possible. The data for our bibliometric analysis came from the WoSCC. To avoid database updates, all the data were downloaded on June 19, 2023. The index terms were: ((TI = (nutri* OR diet)) OR KP = (nutri* OR diet)) AND ((TI = (dement* OR Alzheimer* OR cognit*impair*)) OR KP = (dement* OR Alzheimer* OR cognit*impair*)). Articles in English published from January 1, 2003, to June 30, 2023, were searched with literature type restricted to "article" or "review." Next, two independent researchers manually screened the articles by their titles and abstracts, and eliminated irrelevant articles. Finally, a total of 922 articles were exported to CiteSpace for bibliometric analysis. Figure 1 shows the search strategy in detail. This study did not involve human subjects and/or animals.

### Visual analysis tool—CiteSpace

We chose the 5.7.R2W version of CiteSpace (developed by Professor Chaomei Chen), as the main tool for bibliometric and visual analysis [13]. We saved the document data from the WoSCC as a complete record and exported these data in plain text format (named download_XXX.txt). Before importing the data into CiteSpace, we created a new folder and created four new folders in it, which we named input, output, data, and project. As the time-period selected for the study was January 2003 to June 2023, the time-slice we used was one year [14]. We selected Author, Institution, Country, References, and Keywords as the nodes, and set others as default values. The node size represents the frequency of occurrence or citation, and the node color from cool to warm represents early to recent. In addition, the nodes with purple trim indicate high betweenness centrality, which are commonly considered hotspots or milestones in a field.

## Results

### Number of publications during the last two decades

**Fig. 2:**
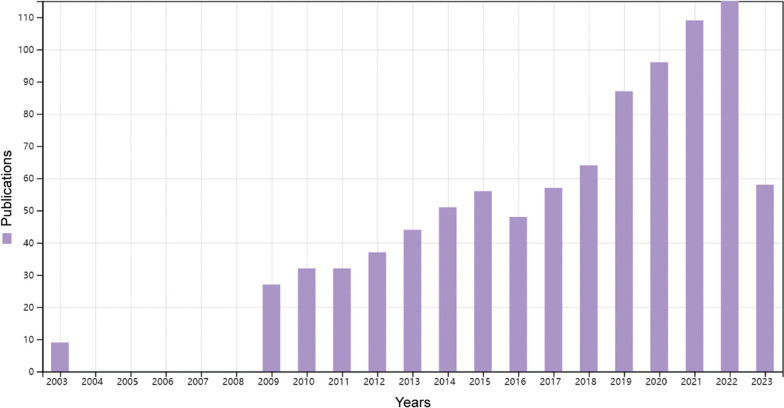
Annual publications chart (from 2003 to 2023)

In total, 922 publications were retrieved through the WOSCC (Fig. 1). The annual number of publications on diet in AD during the past two decades is shown in Fig. 2. There were only 10 publications in 2003, and there were no related publications from 2004 to 2008. From 2009 to 2018, the annual number of publications exhibited a steady growth trend, with an average of 45 publications per year. This area of research entered a period of rapid growth starting in 2019, with an average of 101 publications per year from 2019 to 2022.

### Bibliometric analysis of countries and institutions

**Fig. 3:**
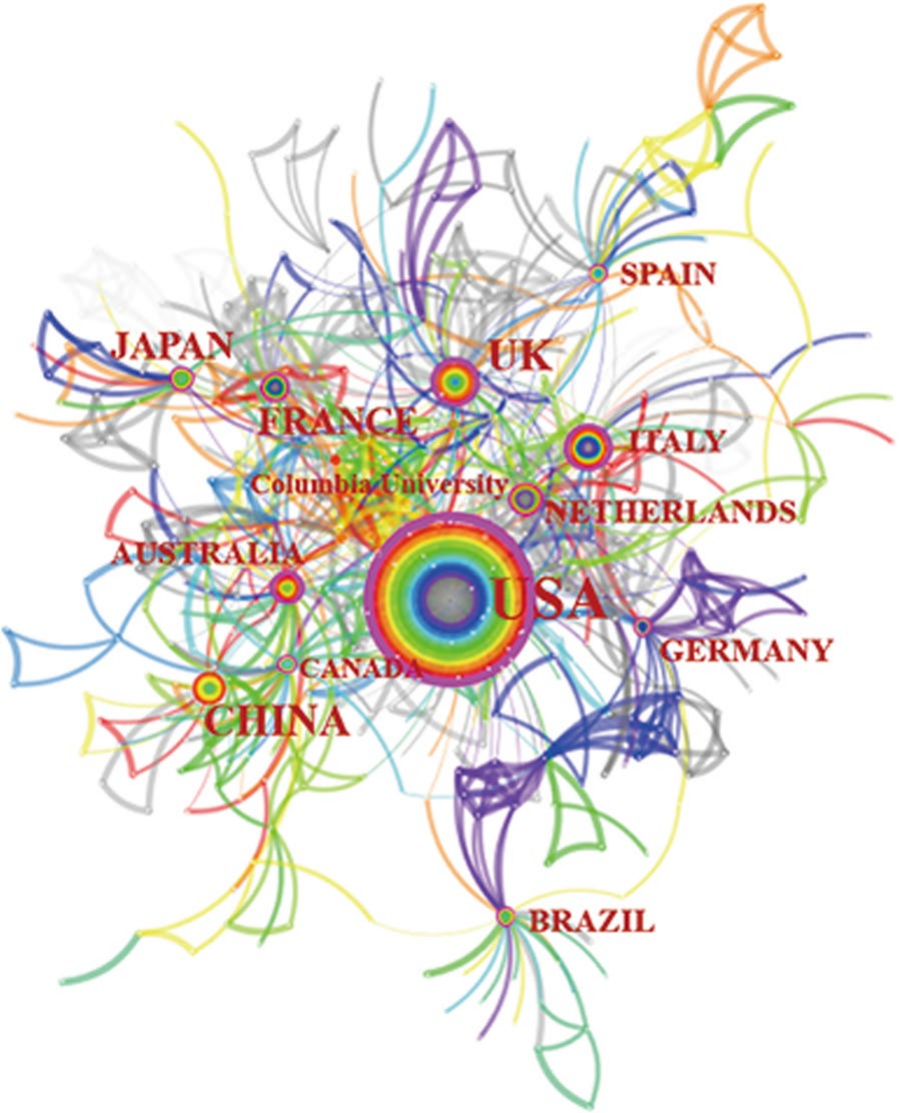
Map of countries and institutions that contributed publications on diet in AD. Nodes represent the 14 countries and 1 institution with the highest contributions. The size of the node indicates the number of publications. The density of the lines between nodes represents the intensity of collaboration between them. AD = Alzheimer’s disease

The top 10 countries and institutions making the greatest contributions to the field are shown in Table 1. These 10 countries accounted for 75.93% of all the publications in the field and the 10 institutions accounted for 11.81% of all the publications. The top 5 countries were the United States of America (USA), the United Kingdom, Italy, Australia, and the People’s Republic of China. The top 5 institutions were Columbia University, Rush University, Aristotle University, Harokopio University, and the National and Kapodistrian University of Athens. As shown in Fig. 3, countries and institutions actively cooperated, with the USA and Columbia University playing leading roles.

**Table 1:** The top 10 countries and institutions with the highest contributions of publications

Rank	Country	*N* (%)	Centrality		Institution	*N* (%)	Centrality
1	United States of America	209 (22.67)		0.75	Columbia University	23 (2.49)	0.03
2	United Kingdom	90 (9.76)		0.35	Rush University	13 (1.41)	0.05
3	Italy	68 (7.38)		0.24	Aristotle University	11 (1.19)	0.00
4	Australia	53 (5.75)		0.20	Harokopio University	11 (1.19)	0.00
5	China	53 (5.75)		0.14	The National and Kapodistrian University of Athens	11 (1.19)	0.01
6	Japan	52 (5.64)		0.17	University College London	10 (1.08)	0.03
7	Netherlands	48 (5.21)		0.21	Boston University	8 (0.87)	0.02
8	France	44 (4.77)		0.17	Athens Alzheimer's Disease-Related Disease Association	8 (0.87)	0.00
9	Canada	43 (4.66)		0.16	Radboud University Nijmegen	7 (0.76)	0.00
10	Germany	40 (4.34)		0.13	Vrije University Amsterdam	7 (0.76)	0.00

### Bibliometric analysis of authors

**Fig. 4:**
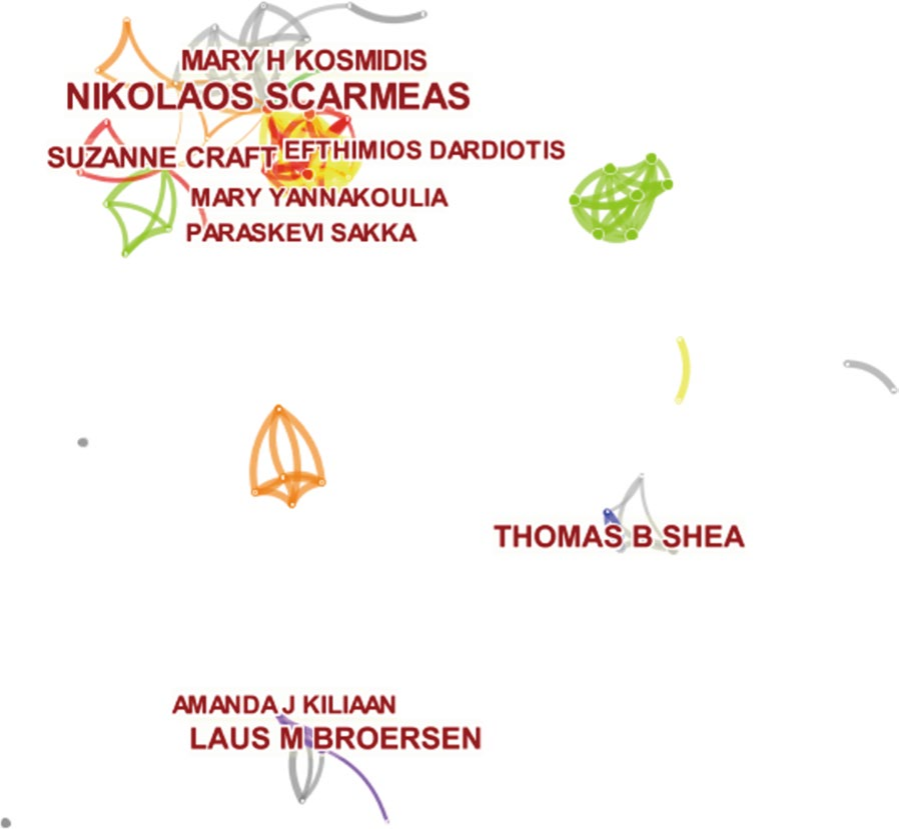
The network of co-authors. Nodes indicate the 9 authors with the highest contributions. The size of the node indicates the number of publications. The density of the lines between nodes represents the intensity of collaboration between them

Table 2 lists the top 9 authors contributing to publications on diet in AD. In total, they contributed 60 studies (6.50%). The most prolific author was Nikolaos Scarmeas (13 studies, 1.41%), followed by Thomas B. Shea (8 studies, 0.87%), Laus M. Broersen (7 studies, 0.76%), Suzanne Craft (6 studies, 0.65%), and Mary H. Kosmidis (6 studies, 0.65%). As shown in Fig. 4, multiple prolific authors collaborated with each other.

**Table 2:** The top 9 authors who contributed to publications on diet in AD

Rank	Author	Count of articles	Centrality
1	Nikolaos Scarmeas	13	0.01
2	Thomas B Shea	8	0.00
3	Laus M Broersen	7	0.00
4	Suzanne Craft	6	0.00
5	Mary H Kosmidis	6	0.00
6	Efthimios Dardiotis	5	0.00
7	Amanda J Kiliaan	5	0.00
8	Paraskevi Sakka	5	0.00
9	Mary Yannakoulia	5	0.00

AD, Alzheimer’s disease

### Bibliometric analysis of co-cited reference

The top 10 co-cited references related to diet in AD are shown in Table 3. The top 5 citations were: (1) Scarmeas et al. [4], who analyzed the protective function of common nutrients, foods, and dietary patterns and called for advanced technologies and biomarkers for making nutritional assessments; (2) Livingston et al. [2], who reported modifiable risk factors that affect the brain (e.g., reducing smoking and managing diabetes and obesity) and also focused on support and end-of-life care for dementia patients; (3) Petersson and Philippou [15], who summarized that the Mediterranean Diet (MeDi) might contribute to better cognitive performance; (4) Scheltens et al. [16], who introduced a randomized, controlled trial, which demonstrated that medicinal food containing phosphatide precursors and cofactors improved memory (i.e., improved performance on delayed verbal recall task) in mild AD patients; and (5) van den Brink et al. [7], who concluded that adherence to the Mediterranean, Dietary Approaches to Stop Hypertension (DASH), or Mediterranean-DASH Intervention for Neurodegenerative Delay (MIND) diets were related to reduced cognitive decline and the incidence rate of AD based on current studies, in which the strongest association was observed for the MIND diet; the MeDi had the second strongest association.

**Table 3:** The details of top 10 most co-cited references

Rank	Title	First author	Year	Journal	Count
1	Nutrition and prevention of cognitive impairment	Scarmeas N	2018	LANCET NEUROL	27
2	Dementia prevention, intervention, and care	Livingston Gill	2017	LANCET	27
3	Mediterranean diet, cognitive function, and dementia: a systematic review of the evidence	Petersson SD	2016	ADV NUTR	18
4	Efficacy of a medical food in mild Alzheimer’s disease: a randomized, controlled trial	Scheltens P	2010	ALZHEIMERS DEMENT	17
5	The Mediterranean, Dietary Approaches to Stop Hypertension (DASH), and Mediterranean-DASH Intervention for Neurodegenerative Delay (MIND) Diets are associated with less cognitive decline and a lower risk of Alzheimer’s disease—a review	van den Brink AC	2019	ADV NUTR	17
6	Association of Mediterranean diet with mild cognitive impairment and Alzheimer's disease: a systematic review and meta-Analysis	Singh B	2014	J ALZHEIMERS DIS	16
7	MIND not Mediterranean diet related to 12-year incidence of cognitive impairment in an Australian longitudinal cohort study	Hosking DE	2019	ALZHEIMERS DEMENT	15
8	Efficacy of Souvenaid in mild Alzheimer’s disease: results from a randomized, controlled trial	Scheltens P	2012	J ALZHEIMERS DIS	15
9	Mediterranean diet and age-related cognitive decline: a randomized clinical trial	Valls-Pedret C	2015	JAMA INTERN MED	15
10	MIND diet associated with reduced incidence of Alzheimer's disease	Morris MC	2015	ALZHEIMERS DEMENT	14

### Bibliometric analysis of research hotspots

#### Co-occurring keywords and keywords cluster

**Fig. 5:**
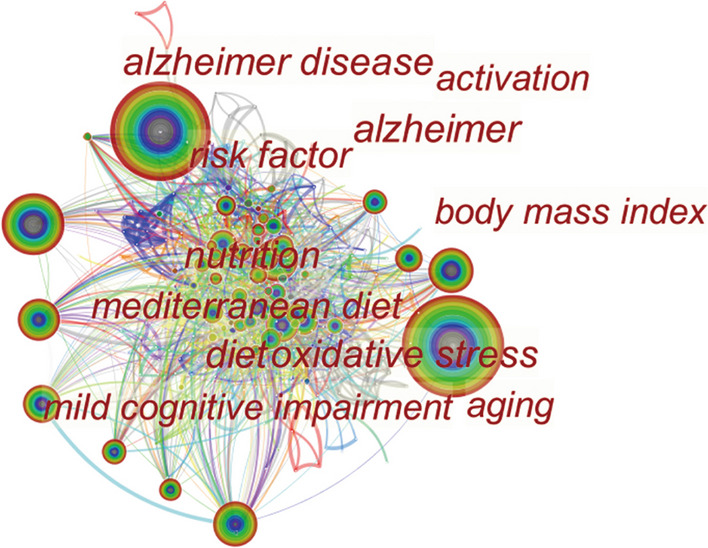
Co-occurring keywords map. Every node represents a keyword, and the lager the node, the higher the frequency of keyword occurrence

Frequency and centrality, respectively, indicate the popularity and importance of keywords in the research field. Co-occurring analysis was used for visually displaying the main research content and hotspots in this field. Table 4 lists the top 10 highest frequency and centrality keywords we found. Figure 5 shows the co-occurring keywords that interconnect with each other. AD, dementia, nutrition, and diet ranked the highest in terms of frequency and centrality. The most popular dietary pattern was the MeDi. The exploration of dietary-related pathological mechanisms mainly involved oxidative stress, apolipoprotein E, and amyloid-beta (Aβ). The most commonly used observational nutrition measure was body mass index. Diet was considered to be one of the modifiable hazard factors for AD, mainly for improving mild cognitive impairment (MCI) and cognitive decline.

**Table 4:** The top 10 high-frequency and centrality keywords for diet in AD

Rank	Count	Keywords	Centrality	Keywords
1	321	Alzheimers disease	0.14	alzheimer
2	281	dementia	0.11	Alzheimer disease
3	132	nutrition	0.10	Diet
4	99	Mediterranean diet	0.10	Oxidative stress
5	97	Risk	0.08	nutrition
6	84	Diet	0.08	Body mass index
7	78	brain	0.08	Apolipoprotein e
8	68	Mild cognitive impairment	0.08	Aging
9	66	Oxidative stress	0.07	Mild cognitive impairment
10	62	Cognitive decline	0.07	A beta

AD, Alzheimer’s disease

**Fig. 6:**
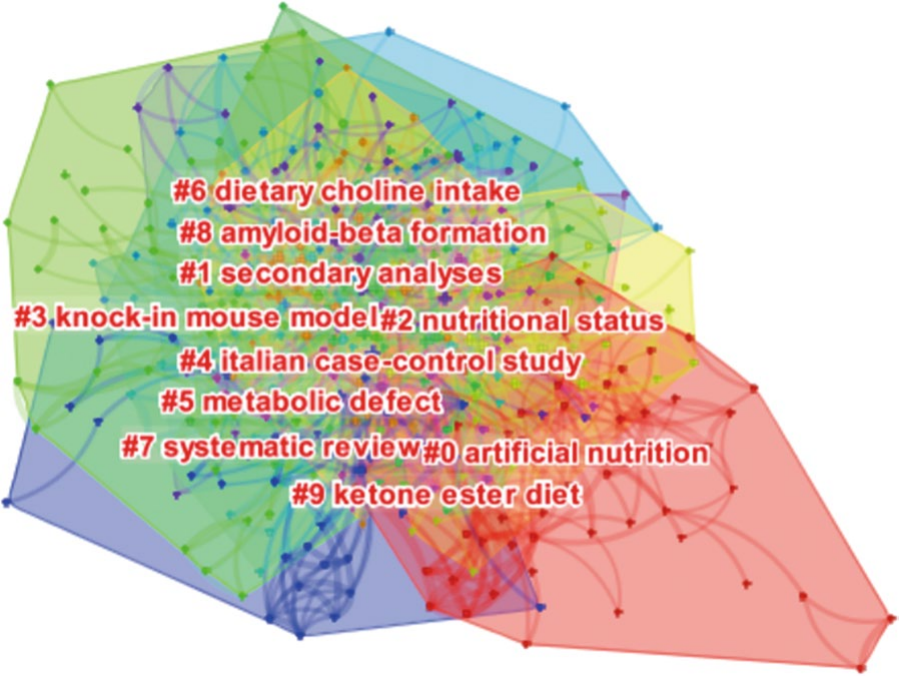
Clustering map of co-occurring keywords. Ten different clusters reflect different research directions

The clustering map of co-occurring keywords is shown in Fig. 6. Different clusters reflect different research directions, which are shown in Table 5. Each cluster had a silhouette value above 0.6, illustrating the significant effectiveness of the clustering structure.

**Table 5:** Ten clusters of keywords for diet in AD

Cluster ID	Silhouette	Label (LLR)	Included keywords (top 5)	Mean (year)
0	0.865	Artificial nutrition	dementia; population; mortality; neurodegeneration; nursing home resident	2012
1	0.725	Secondary analyses	brain; memory; cognitive impairment; obesity; docosahexaenoic acid	2013
2	0.755	Nutritional status	Older adult; decline; malnutrition; disease; weight to	2015
3	0.743	Knock-in mouse model	Mouse model; a beta; homocysteine; mice; activation	2016
4	0.839	Italian case–control study	Mediterranean diet; diet; mild cognitive impairment; risk factor; cognition	2012
5	0.784	Metabolic defect	Cognitive function; inflammation; performance; tau; nutrient	2017
6	0.679	Dietary choline intake	alzheimer; metabolism; beta; dietary pattern; brain insulin resistance	2016
7	0.899	Systematic review	Alzheimers disease; oxidative stress; Alzheimer disease; vitamin e; amyloid-beta	2010
8	0.785	Amyloid-beta formation	nutrition; risk; cognitive decline; insulin resistance; aging	2011
9	0.682	Ketone ester diet	health; impairment; association; elderly; mini mental state	2011

AD, Alzheimer’s disease

#### Keywords with citation bursts

**Fig. 7:**
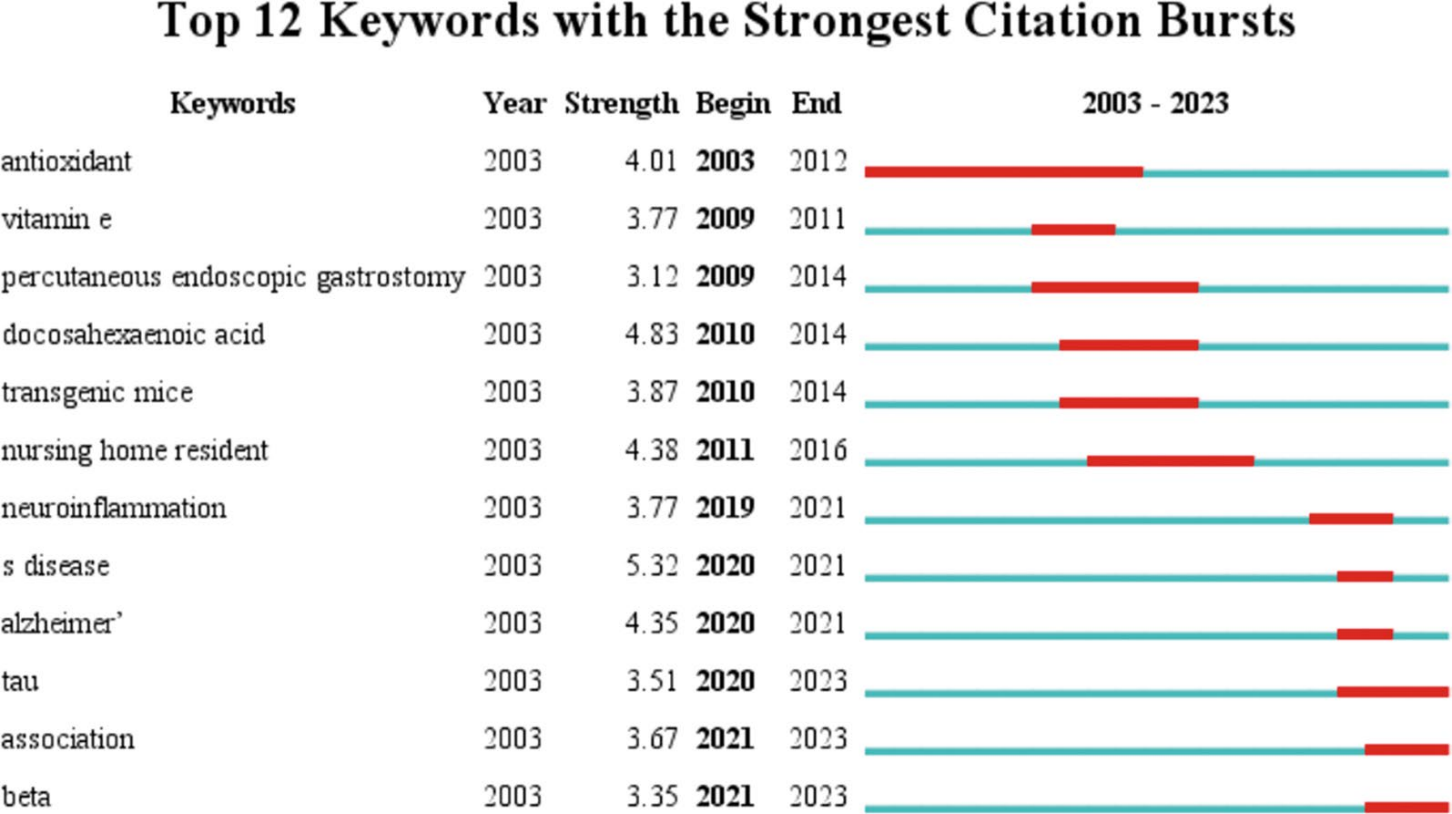
Top 12 keywords with the strongest citation bursts over two decades. The red line represents the keyword burst duration, and the blue line represents the time interval

Figure 7 displays the top 12 keywords with the strongest citation bursts. The keyword antioxidant appeared early in 2003 and lasted until 2012, indicating what the research frontier was a decade ago. Every year from 2019 to 2021, there were new keyword bursts (i.e., neuroinflammation, AD, tau, association, and beta), indicating a continuous increase in research enthusiasm for the field of diet in AD (which is consistent with the rapid growth period of publications in Fig. 2). In recent years, tau protein, association, and Aβ have become major research frontiers.

## Discussion

### Summary of findings

This study elucidated the research hotspots and trends related to the effect of diet on AD over the past two decades using CiteSpace. The annual number of publications to some degree indicated the research enthusiasm in this field. Our study indicated a certain breakthrough in research results since 2019. The USA and Columbia University showed great interest in this field and were the country and institution with the most publications. Most of the other countries with high contributions to the field included Australia and several European countries, while Asian countries only included China and Japan. It is worth noting that dietary patterns vary with different environmental and cultural characteristics. Some countries and regions (e.g., South America and Southeast Asia) that were not mentioned in this study may not have received attention due to insufficient research data or underdeveloped medical technology. Comparing the top 10 co-cited references and the top 9 authors, Nikolaos Scarmeas was not only the most prolific author, but he also published an article with the most citations. In addition, Scheltens contributed two highly cited articles, indicating the high quality of his articles.

### Research hotspots

#### Mediterranean diet (MeDi)

The traditional MeDi is represented by the diet of Greece, Spain, France, and some other European countries. It is a dietary style dominated by vegetables, fruits, fish, cereals, beans, and olive oil and is also the most popular dietary pattern in our study. Early in 2006, an observational study by Scarmeas et al. found that among 2,258 community-based individuals without dementia in New York, the greater their adherence to the MeDi was, the smaller their risk of AD was during the average 4-year observation period [17]. Over the past decade, with the increase of relevant observational studies, clinical trials, and systematic reviews, the exploration of MeDi-related brain functions has gradually deepened. A randomized clinical trial conducted with 447 healthy volunteers in Barcelona (average age = 66.9 years) demonstrated that the MeDi with extra-virgin olive oil and the MeDi with mixed nuts both performed better than the control diet in follow-up cognitive examinations (median = 4.1 years) [18]. In Roy J. Hardman’s systematic review of longitudinal and prospective trials [19], 18 articles related to the MeDi and cognition met the inclusion criteria, among which 13 articles indicated that the high adherence to the MeDi was related to slowing cognitive decline, reducing the probability of AD progression, and improving cognitive function. Another five articles did not find a relationship between the MeDi and cognitive performance (but whole grain intake and the relative proportions of unsaturated and saturated fat intake were independently related to cognitive function [20]). The beneficial effects of the MeDi may not be limited to European countries or certain regions. Studies conducted in the USA, Spain, and Australia have shown a correlation between adherence to the MeDi and reduced risk of AD and MCI [3, 21, 22]; there is still a lack of the MeDi applied research on Asian populations. The MeDi should not be thought of just in terms of Mediterranean cuisine. Bere and Brug believed that it was more appropriate to form a diet model that was more suitable for local people according to its dietary characteristics, cultural heritage, and environmental factors than to mechanically promoting the MeDi on a large scale [23]. Asian dietary patterns are more similar to the MeDi in terms of food selection, compared to the Western diet. A prospective cohort study confirmed that the Japanese diet (rich in soybean and soybean products, vegetables, algae, and milk and dairy products, with a small amount of rice) was related to a reduced risk of AD [24]. The DASH diet is also a dietary pattern similar to the MeDi in terms of food composition, and it has been shown to have a positive impact on hypertension and cardiovascular disease risk factors [7]. Based on the MeDi and DASH diets, the MIND diet was developed by combining the advantages of both of them. The MIND diet has been found to be associated with reduced cognitive decline and incidence rate of AD [25]. Some studies have reported the strongest association with the MIND diet followed by the MeDi [7, 26].

The MeDi's neuroprotective effect may be attributable to a number of food ingredients, such as polyunsaturated fatty acid in fish, mono unsaturated fat in olive oil, and polyphenols and antioxidant compounds in fruits, vegetables, and wine. Angeloni et al. provided a new perspective on the effect of extra-virgin olive oil in their review. Olive oil increased brain glutathione levels to help reduce oxidative stress, and long-term olive oil consumption from a young age led to an enhanced Aβ clearance pathway and reduced the brain’s production of Aβ [27]. In addition, people who follow the MeDi have specific characteristics of intestinal microbiota, which may enhance neuroprotective action via the microbiota-gut-brain axis [28]. The MeDi may also affect the morbidity of patients with MCI and AD by protecting the cardiovascular system, which has been confirmed by systematic reviews and meta-analyses [29, 30]. The mechanisms of action of the MeDi are constantly being revealed, including but not limited to the regulation of insulin resistance and inflammation [31], the activity of the brain–gut axis, and the impact of intestinal microbiota on brain function, which still needs to be clarified by larger-scale animal testing and human studies.

Over the past two decades, the results of a large number of studies have supported the relationship of the MeDi with AD and MCI, but most of the subjects in these observational and clinical studies were more than sixty years old. Whether earlier dietary interventions can further reduce the risk of AD requires longer follow-up periods to verify the results. Moreover, the use of large, long-term clinical trials that are well-powered and employ biomarkers is one of the major directions of future research.

#### Oxidative stress

Oxidative stress is an important player in the pathophysiologic process of AD [32]. Currently, it is believed that the pathological manifestations of AD mainly include neurofibrillary tangles and the accumulation of Aβ peptide in the brain, which might be involved in complex molecular reactions in neurons and the results of the oxidative stress response in the aging brain [33]. Therefore, antioxidant theory has become one of the popular research topics on the role of diet in AD. Vegetables and fruits contain rich antioxidant elements, such as water-soluble vitamin C and fat-soluble vitamins A, D, E, and K, the absence of which has been related to cognitive decline in senior citizens [9]. Mild-to-moderate alcohol consumption is related to reduced incidence rates of AD and dementia [4, 10]. The polyphenols, anthocyanins, resveratrol, and gallic acid in red wine may help prevent the oxidative damage observed in AD [10]. In addition to a single kind of food, the effect of the MeDi and ketogenic diet (KD) on AD is considered to be related to antioxidation [34, 35], which may be the accumulation of food effects or a synergistic effect among foods. Neuropathological research on AD is still ongoing, and the specific molecular expression of oxidative stress is becoming clearer. The antioxidant mechanisms of more nutrients are waiting to be discovered. Another research question worth noting is whether the antioxidant effects of certain foods or nutrients related to AD depend on a specific dose range. To explain the role of nutrients better, research should also examine their appropriate amounts.

#### Souvenaid (fortasyn connect)

Souvenaid is a medicinal drink used to support early memory function in patients with AD. It can be regarded as an extension of the MeDi. The main component of this product is a unique nutrient combination called Fortasyn Connect (FC), containing a mixture of precursors and cofactors, viz. docosahexaenoic acid, eicosapentaenoic acid, uridine monophosphate, choline, phospholipids, folic acid, vitamins B6, B12, C, and E, and selenium, which help support the formation of synaptic nerves [36, 37]. In a 24-week, randomized, controlled, double-blinded, parallel-group, multi-country trial, Scheltens et al. confirmed that Souvenaid changed synaptic activity in mild AD patients without medical treatment [38]. Broersen et al. found decreased Aβ levels in the brain and amyloid plaque levels in the hippocampus, as well as a reduction in the disintegrative degeneration of the neocortex in Aβ PP/PS1 transgenic mice receiving a FC diet [37]. Therefore, as the food for special medical purposes (FSMP) with the most abundant research evidence, Souvenaid gradually strengthens its clinical promotion. Experts agree that patients with early AD and MCI should take a daily supplement of Souvenaid [36], but Souvenaid should not be recommended to patients who have middle and late AD, lactose intolerance, or an allergy to fish, soy, milk protein, or vegan diets. Longer follow-up experiments are needed in the future to explore the effect of a FC diet on cognitive function, as well as to verify the need for lifelong supplementation with Souvenaid.

### Research trends

#### Effectiveness of various dietary patterns

Over the past two decades, experts around the world have explored the effects of various nutrients and dietary patterns on cognitive function and the incidence rate of AD. Humans do not consume only a single type of food; thus, more attention should be paid to comprehensive dietary patterns, which include the MeDi, KD, the DASH, the MIND, and vegan diets, as well as whole-food diets, etc. Differences in sample size, tracking time, experimental methods, and evaluation standards have produced different research results, which cannot lead to definitive and unified conclusion. The introduction of new food combinations and the validation of the effect of various dietary patterns with advanced technology are expected to be some of the major trends during the next decade.

#### Mechanisms of dietary interventions

Neuroinflammation, tau protein, and Aβ-related topics have burst since 2019 (Fig. 7), indicating that the exploration of the mechanism of diet on AD is gradually approaching to neuroinflammation and biomarkers. McGrattan et al. [6] summarized that long-chain omega-3 fatty acids from fish contributed to eliminate inflammation by reducing the expression of pro-inflammatory cytokines in microglia. A pilot study confirmed that folic acid supplementation reduced the levels of peripheral inflammatory cytokines and improved cognition with the combination of Donepezil for 6 months [39]. de Wilde et al. [40] inferred from a rats experiment that FC diet might protect cholinergic neurons by improving membrane integrity and limiting the membrane-binding and membrane-disrupting properties of Aβ 42. As for comprehensive dietary patterns, KD decreases systemic inflammation by increasing production of anti-inflammatory proteins and decreasing inflammatory response to toxic free radicals [41]. Moreover, the anti-inflammatory and antioxidant effects of foods may explain how the MeDi is related to reduced Aβ 42/40 ratio and phosphorylated tau 181 in the cerebrospinal fluid [42]. On the contrary, Western diet contains a large amount of saturated fatty acids and simple sugars increase the activity of peripheral pro-inflammatory cytokines and results in a systemic low-grade inflammatory state, which eventually leads to the progression of AD [43]. Diets also affect biomarker levels by regulating the type and abundance of gut microbiota. A clinical cohort study indicated that the modified Mediterranean-Ketogenic diet (MMKD) changed the levels of AD-related biomarkers by affecting gut microbiota with the production of short-chain fatty acid. For example, MMKD increased phylum *Tenericutes*, which correlated negatively with the level of Aβ 42 in MCI patients. And family *Ruminococcaceae* was positively correlated with phosphorylated tau 181 in cognitively normal participants after MMKD[44]. Similarly, dietary patterns rich in insoluble fiber, including the MeDi, alters gut microbiota composition (decreased Firmicutes and increased Bacteroidetes) and produces high levels of short-chain fatty acids, which have been illustrated to suppress the development of several inflammatory disease [45]. With the continuous discovery of AD-related biomarkers (e.g., Aβ, tau protein, neuroinflammation, and reactive astrogliosis [46]), analyzing pathological mechanisms of foods on AD using biomarkers has become a cutting-edge direction in this research field.

#### Supplementation with refined nutrients

Compared to dietary patterns, oral supplement of FSMP (e.g., Souvenaid and Ketogenic beverages) controls the dosage more accurately and is more convenient in practical operation (adding them after three meals [47]). Parsons et al.’s research indicated that oral nutrient supplementation improved the quality of life and the nutritional intake of malnourished residents in sanatoriums more effectively than simple dietary changes did [48]. However, supplements of FSMP take some time to demonstrate their therapeutic effects, and the specific time it takes varies from person to person. Currently, evidence on different types of FSMP supplements and their effects is limited and conclusions are inconsistent. Supplementation with refined nutrients deserves further attention.

### Strengths and limitations

As far as we know, this is the first study on bibliometric and visual analysis using CiteSpace to describe the research status, hotspots, and trends regarding the role of diet in AD. Detailed and objective data analysis showed how the number and focus of published articles on diet and AD have changed over the past 20 years. However, the study also has some shortcomings. First, we only retrieved articles from the WOSCC by titles and keywords; hence, we may have overlooked research data that were not included in the WOSCC. Second, we set a 20-year time limit and an English language limit in the search criteria, so we were not very familiar with studies from earlier periods and we did not examine the origin of food interventions in AD. We also omitted published studies that were not in English. These shortcomings may have led to incomplete research content.

## Conclusions

This study visually presents the development of the role of diet in AD over the past two decades using CiteSpace. There is a certain degree of collaboration among the countries, institutions, and some authors. Still, talent exchange and regional cooperation are recommended. Research hotspots include dietary patterns (the MeDi), intervention mechanisms (oxidative stress), and FSMP (Souvenaid). Integrating the research directions of the major authors and high-impact articles, we predict that the main research trends in the future will be assessing the effectiveness of various dietary patterns and exploring the mechanisms of dietary interventions using biomarkers, represented by Aβ and tau protein, and supplementation with refined nutrients. Hopefully, with the cooperation of the elderly, neurologists, and dietitians, it may possible to provide a new method with higher acceptance and greater operability to promote the aging brain health through dietary interventions.

## Data Availability

All data generated or analyzed during this study are included in this published article; further inquiries can be directed to the corresponding author on reasonable request.

## Abbreviations

AD: Alzheimer’s disease
WOSCC: Web of Science Core Collection
USA: United States of America
MeDi: Mediterranean diet
DASH: Dietary Approaches to Stop Hypertension
MIND: Mediterranean-DASH Intervention for Neurodegenerative Delay
Aβ: Amyloid-beta
MCI: Mild cognitive impairment
KD: Ketogenic diet
FC: Fortasyn connect
FSMP: Food for Special Medical Purposes
MMKD: Modified Mediterranean-ketogenic diet
